# Supplementary material for: Genetic Evaluation in a Cohort of 126 Dutch Pulmonary Arterial Hypertension Patients
Source: Genes (Basel). 2020 Oct 13;11(10):1191. doi: 10.3390/genes11101191 (PMC7602048; doi:10.3390/genes11101191)
Supplement: Supplementary file 1 [file genes-11-01191-s001.pdf]

Supplementary material

Supplementary Material S1

Detailed information on the genetic analysis methods

Genes associated with PAH were analyzed by a WES-based virtual gene panel. Libraries were prepared with the Kapa HTP kit (Illumina), capture was performed with the SeqCap EZ MedExome kit (Roche NimbleGen). Sequencing was performed on an Illumina HiSeq4000 HTv4 with paired-end, 125 bp reads. The read alignment to GRCh37 (hg19) and variant calling were conducted with a pipeline based on the Burrows-Wheeler Aligner BWA-MEM 0.7 and the Genome Analysis Toolkit (GATK) 3.3.0. VCF files were filtered for variants in the genes of interest in Alissa (Agilent Technologies) and variant classification was performed using Alamut Visual according to ACGS/VKGL guidelines (1)

References

1. Wallis, Y.; Payne, S.; McAnulty, C.; Bodmer, D.; Sistermans, E.; Robertson, K.; Moore, D.; Abbs, S.; Deans, Z.; Devereau, A.; et al. Practice guidelines for the evaluation of pathogenicity and the reporting of sequence variants in clinical molecular genetics. Association for Clinical Genetic Science and the Dutch Society of Clinical Genetic Laboratory Specialists. 2013.

Supplementary Material S2

Table S2. Overview of the detected class 3 variants in unrelated PAH patients.

Gene	Nucleotide Change	Amino Acid Change	Pathogenicity
TBX4	c.167C>T	p.(Ala56Val)	Class 3
TBX4	c.1145A>C	p.(Tyr382Ser)	Class 3
NOTCH3	c.5551C>T	p.(Arg1851Cys)	Class 3
NOTCH3	c.1112del	p.(Pro426Leu)	Class 3
NOTCH3	c.2803A>C	p.(Asn935His)	Class 3
NOTCH3	c.2890G>C	p.(Asp964His)	Class 3
BMPR2	c.848_849insATA	p.(Pro283_Asn284insTyr)	Class 3
BMPR2	c.1178A>G	p.(Asn393Ser)	Class 3[a]
BMPR2	c.2729G>A	p.(Cys910Tyr)	Class 3
FOXF1	c.427G>C	p.(Gly143Arg)	Class 3
FOXF1	c.613G>C	p.(Gly205Arg)	Class 3[a]
FOXF1	c.1021C>G	p.(Arg341Gly)	Class 3

Reference sequences: TBX4 NM_018488.2; NOTCH3 NM_000435.3; BMPR2 NM_001204.6; FOXF1 NM_001451.3

[a]These variants were found in one patient
